# Terrestrial Plants Evolve Highly Assembled Photosystem Complexes in Adaptation to Light Shifts

**DOI:** 10.3389/fpls.2018.01811

**Published:** 2018-12-19

**Authors:** Yang-Er Chen, Yan-Qiu Su, Hao-Tian Mao, Nan Wu, Feng Zhu, Ming Yuan, Zhong-Wei Zhang, Wen-Juan Liu, Shu Yuan

**Affiliations:** ^1^College of Life Sciences, Sichuan Agricultural University, Ya’an, China; ^2^College of Life Science, Sichuan University, Chengdu, China; ^3^College of Horticulture and Plant Protection, Yangzhou University, Yangzhou, China; ^4^College of Resources, Sichuan Agricultural University, Chengdu, China; ^5^Center of Analysis and Testing, Sichuan Academy of Agricultural Sciences, Chengdu, China

**Keywords:** LHCII assembly, nonphotochemical quenching, phosphorylation, photosystem evolution, PS supercomplexes, state transition

## Abstract

It has been known that PSI and PSII supercomplexes are involved in the linear and cyclic electron transfer, dynamics of light capture, and the repair cycle of PSII under environmental stresses. However, evolutions of photosystem (PS) complexes from evolutionarily divergent species are largely unknown. Here, we improved the blue native polyacrylamide gel electrophoresis (BN-PAGE) separation method and successfully separated PS complexes from all terrestrial plants. It is well known that reversible D1 protein phosphorylation is an important protective mechanism against oxidative damages to chloroplasts through the PSII photoinhibition-repair cycle. The results indicate that antibody-detectable phosphorylation of D1 protein is the latest event in the evolution of PS protein phosphorylation and occurs exclusively in seed plants. Compared to angiosperms, other terrestrial plant species presented much lower contents of PS supercomplexes. The amount of light-harvesting complexes II (LHCII) trimers was higher than that of LHCII monomers in angiosperms, whereas it was opposite in gymnosperms, pteridophytes, and bryophytes. LHCII assembly may be one of the evolutionary characteristics of vascular plants. *In vivo* chloroplast fluorescence measurements indicated that lower plants (bryophytes especially) showed slower changes in state transition and nonphotochemical quenching (NPQ) in response to light shifts. Therefore, the evolution of PS supercomplexes may be correlated with their acclimations to environments.

## Introduction

Chloroplasts are plant cell organelles in which photosynthesis and other biosynthetic pathways occur. The primary reactions of plant and algal photosynthesis occur in thylakoid membranes of the chloroplasts. There are four major multimeric protein complexes in thylakoid membranes: photosystem I (PSI), photosystem II (PSII), the cytochrome *b*_6_/*f* complex, and the ATP synthase (Caffarri et al., 2009; Nevo et al., 2012; Nelson and Junge, 2015). These complexes contain at least 70 different proteins that work together ultimately to produce ATP and NADPH as products. In addition, the thylakoid membrane also harbors light-harvesting complexes (LHC) and transporters of electrons (Nelson and Junge, 2015).

It has been known that PSI and PSII supercomplexes and subcomplexes are involved in linear and cyclic electron transfer, dynamics of light capture, and the repair cycle of PSII under environmental stresses (Chen et al., 2013, 2016a, 2017; van Bezouwen et al., 2017). For example, under light stress, monomerization of PSII-LHCII supercomplexes and the migration of damaged PSII cores to unstacked stroma of thylakoid membranes occur (Baena-Gonzalez et al., 1999; Yoshioka-Nishimura, 2016). Our recent studies have demonstrated that drought stress or high light and high temperature co-stress results in the rapid disassembly of PSII-LHCII supercomplexes and LHCII assemblies (Chen et al., 2016a, 2017). In addition, PSI-NDH (NAD(P)H dehydrogenase) and PSI-LHCII complexes were shown to be involved with NDH-dependent cyclic electron transfer-specific megacomplexes and state transition in *Arabidopsis thaliana*, respectively (Peng et al., 2009; Armbruster et al., 2013). However, it is unknown whether thylakoid membrane protein complexes in evolutionarily divergent organisms present evolutionary differences under natural conditions.

To understand the composition and dynamic function of thylakoid protein complexes in different plant species, it is essential to develop techniques allowing the reliable separation of these protein complexes. The blue native polyacrylamide gel electrophoresis (BN-PAGE) has become an indispensable tool for the analysis of respiratory and photosynthetic protein complexes under native conditions (Swamy et al., 2006; Järvi et al., 2011). However, the thylakoid membrane complexes in gymnosperms and ferns have not been separated successfully by traditional BN-PAGE. In the present experiment, we developed an improved BN-PAGE protocol for the separation of the thylakoid membrane protein complexes from different plant species. Using this improved technique, we have found differences in the amount or composition of thylakoid protein complexes in evolutionarily divergent species with oxygenic photosynthesis.

## Materials and Methods

### Plant Materials and Growth Conditions

*Arabidopsis thaliana* (ecotype Col-0), wheat (*Triticum aestivum* L. cv. Chuanmai 42), tomato (*Solanum lycopersicum* Mill. cv. Zhongza No. 9), rice (*Oryza sativa* L. cv. Wuyu21), maize (*Zea mays* L.), and soybean (*Glycine max* cv. ZH13) seedlings were grown for 4 weeks in a sunlit greenhouse at a relative humidity of 60 ± 5% and with day/night temperature of 28/20°C using a 12/12-h light/dark cycle under a light intensity of 100 μmol photons m^−2^ s^−1^. *Pinus massoniana*, *Taxus chinensis*, *Ginkgo biloba*, *Nephrolepis auriculata*, *Alsophila spinulosa*, *Lycopodium squarrosum*, *Hylocomium splendens*, *Physcomitrella patens*, and *Marchantia polymorpha* grown in natural conditions were collected in Sichuan province, China. For these plants, green and young branches or seedlings (current year leaves) were selected. After one day acclimation in the greenhouse (under a 12/12-h light/dark cycle of the light intensity of 100 μmol photons m^−2^ s^−1^), the leaves or plants were harvested for chloroplasts isolation or thylakoid membrane extraction.

### Thylakoid Isolation

Thylakoid membrane isolation was performed in a cold room under dim light according to the previous method (Chen et al., 2016b). For protein phosphorylation analyses, 10 mM sodium fluoride (NaF) was added to all buffers to inhibit protein dephosphorylation during thylakoid isolation. Finally, isolated thylakoid membranes were resuspended in a small aliquot of the storage buffer at a final concentration of at least 1 mg chlorophyll (Chl) ml^−1^. The chlorophyll concentration of the thylakoid samples was measured according to the previous method (Porra et al., 1989). The samples were rapidly stored at −80°C until further analysis. The same protocol was followed for thylakoid membrane isolation for all species utilized in the current study.

### Oxygen Evolution and DCPIP Photoreduction Measurement

The oxygen-evolving activity of thylakoid samples was determined using a Clark-type electrode (Hansatech, Norfolk, United Kingdom) in a reaction medium that consisted of 25 mM Hepes (pH 7.6), 0.2 M sucrose, 10 mM NaCl, and 5 mM CaCl_2_ together with 0.25 mM phenyl-p-benzoquinone (PpBQ) as the artificial electron acceptor (García-Cerdán et al., 2009). The measurements were performed at 20°C under saturating light according to the instructions provided by the manufacturer. 2,6-dichlorophenol indophenol (DCPIP) photoreduction was determined spectrophotometrically according to the previous method (Tang and Satoh, 1985). The components of the reaction mixture were 50 mM MES-NaOH (pH 7.5), 10 mM NaCl, 60 mM DCPIP, 2 mM MgCl_2_, and 40 mg mL^−1^ thylakoid final chlorophylls concentration.

### SDS-PAGE and Western Blot Analysis

Thylakoid membrane proteins were separated either by 14% SDS-PAGE (Laemmli, 1970) with 6 M urea or 16% Tricine SDS-PAGE (Schägger, 2006) for better resolution of low-molecular-mass proteins. About 1 μg of total Chl was loaded for each sample. After electrophoresis, the proteins were visualized by Coomassie Brilliant Blue R staining or were transferred onto a polyvinylidene difluoride (PVDF) membrane (Immobilon, Millipore, Darmstadt, Germany). Then, the membranes were blocked with 5% skim milk. Thylakoid proteins were immunodetected with specific antibodies. For the protein phosphorylation status assay, antiphosphothreonine antibodies purchased from New England Biolabs (Cell Signaling, Ipswich, MA, USA) were used, and PVDF membranes were blocked with 5% BSA (Sigma Chemical Co., St. Louis, MO, USA). Then, the membranes were incubated with horseradish peroxidase-conjugated secondary antibody (Bio-Rad Corp. Hercules, CA, USA) and developed using a chemiluminescent detection system (ECL, GE Healthcare, Buckinghamshire, United Kingdom). Quantity One software (Bio-Rad Corp. Hercules, CA, USA) was used for the quantification of protein.

### BN-PAGE Analysis

BN-PAGE gels were prepared in accordance with previous methods (Swamy et al., 2006; Järvi et al., 2011). The optimal separation of the thylakoid membrane protein complexes by BN-PAGE was developed using a gradient of 5–12.5% acrylamide in the separation gel and 4% acrylamide in the stacking gel.

BN-PAGE was carried out as described previously (Järvi et al., 2011), with minor modifications as follows. For solubilization and loading onto BN gels, the amounts of thylakoid membranes containing 20 μg of Chl were used to clearly find weak bands. The thylakoid samples were washed once with ice-cold resuspension buffer [25 mM Bis/Tris HCl (pH 7.0), 20% (w/v) glycerol]. Then, the pellets were resuspended in 20 volumes of resuspension buffer to a Chl concentration of 1.0 mg ml^−1^. An equal volume (20 μl) of *n*-dodecyl-β-D-maltoside (Sigma) solution (diluted in resuspension buffer) was added to a final concentration of 0.5 to 4.0% (w/v). The thylakoid membranes were then solubilized for 5 min on ice by continuous gentle mixing in darkness. The mixture was centrifuged at 18,000 *g* for 20 min at 4°C to remove the insoluble material. After centrifugation, the supernatant was supplemented with 1/10 volume of sample buffer [100 mM Bis Tris/HCl (pH 7.0), 0.5 M amino-*n*-caproic acid, 30% (w/v) sucrose, and 50 mg ml^−1^ Serva Blue G250]. The quantitative analysis of thylakoid membrane complexes was performed using Quantity One software (Bio-Rad Corp. Hercules, CA, USA).

### 2-D BN/SDS-PAGE Analysis

For the separation of proteins in the second dimension, the gel strips from the first-dimension BN-PAGE were cut off and immediately incubated in 10 ml of Laemmli buffer [138 mM Tris/HCl (pH 6.8), 6 M urea, 22.2% (v/v) glycerol, 4.3% (w/v) SDS, and 5% (v/v) 2-mercaptoethanol] (Laemmli, 1970) for 1 h with gentle shaking at room temperature (21°C). Afterwards, the gel strips were placed on the top of the SDS-PAGE gel containing 15% acrylamide and 6 M urea without 4% stacking gel and subsequently sealed with 0.5% agarose in SDS-PAGE running buffer. After electrophoresis, the proteins were visualized by silver staining (Chevallet et al., 2006).

### *In vivo* Chloroplast Fluorescence Measurements

Functional chloroplasts isolated from 15 plant species were dissolved immediately before analysis in an optimized hypotonic buffer containing 100 mM sorbitol, 5 mM MgCl_2_, 10mM NaCl, 20 mM KCl, 30 mM HEPES, and 0.03% (w/v) agarose (40 mg mL^−1^ chloroplasts final chlorophylls concentration) (Betterle et al., 2015). For fluorescence measurements on functional chloroplasts, final 0.05% (w/v) agarose was added to avoid chloroplast sedimentation (Betterle et al., 2015). Fv/Fm, ΦPSII, and qP were calculated according to the previous method (Maxwell and Johnson, 2000). ΦPSI and ΦPSI (ND) was expressed as the previous method indicated (Klughammer and Schreiber, 1994).

State transition experiments were performed on leaves according to established protocols (Bellafiore et al., 2005). Preferential PSII excitation was provided by illumination with red light (127 μmol photons m^−2^ s^−1^), and excitation of PSI was achieved using far-red light from a light-emitting diode light source applied for 900 s simultaneously with red light.

NPQ was measured through chlorophyll fluorescence on functional chloroplasts at room temperature with a PAM 101 fluorimeter (Dual-PAM-100, Heinz Walz GmbH, Effeltrich, Germany), a saturating light pulse of 3,000 μmol photons m^−2^ s^−1^ for 0.8 s, and actinic red light of 1,000 μmol photons m^−2^ s^−1^ supplied by the fluorimeter (Betterle et al., 2015).

### Transmission Electron Microscopy Observation

Thylakoid ultrastructure was analyzed according to Liu et al. (2009). Transverse section of the leaf samples were fixed with 3% glutaraldehyde in 0.1 M sodium cacodylate buffer (pH 6.9) overnight at 4°C, then fixed with 1% osmium tetroxide, dehydrated with acetone, and embedded in Epon 812. Thin sections were cut with an ultramicrotome (Ultracut F-701704, Reichert-Jung, Reichert, Austria) and were negatively stained with 2% uranyl acetate on glow-discharged carbon-coated copper grids. Electron microscopy was performed using a TEM H600 electron microscope (Hitachi, Midland, ON, Canada) operating at 100 kV.

### Statistical Analysis

The data analysis was performed using the statistical software SPSS 19.0 (IBM, Chicago, IL). The results were shown as the means ± standard deviations (SD) from three independent replicates, and the Duncan’s multiplication range test was adopted. Differences of the mean values among treatments were marked to be statistically significant when *p* < 0.05.

## Results

### Phosphorylation Levels of Thylakoid Proteins

**Figure 1:**
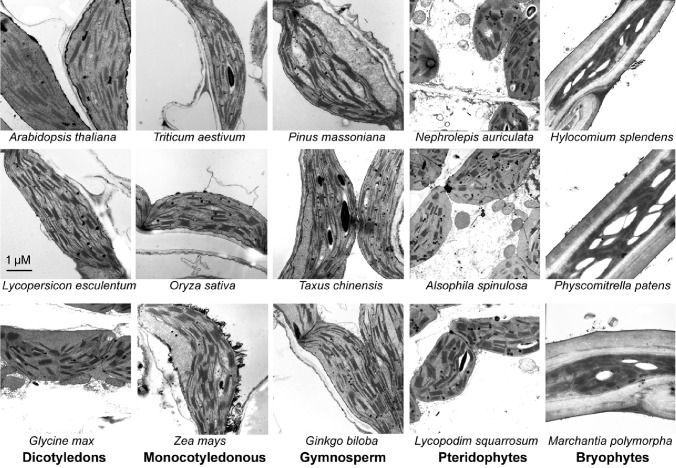
Transmission electron microscope analysis of chloroplasts from 15 plant species. Some of the plants were grown for 4 weeks in a sunlit greenhouse with day/night temperature of 28/20°C using a 12/12-h light/dark cycle under a light intensity of 100 μmol photons m^−2^ s^−1^. The other plants grown in natural conditions were collected in Sichuan province, China. For the plants grown in natural conditions, green and young branches or seedlings were selected. After one day acclimation in the greenhouse (under a 12/12-h light/dark cycle of the light intensity of 100 μmol photons m^−2^ s^−1^), the leaves or plants were harvested for thylakoid ultrastructure observation.

To verify the plant samples we collected are grown in good growth conditions, their thylakoid ultrastructures were observed with a transmission electron microscopy. Although moss chloroplasts show different shapes, there are no obvious changes in grana stacking among all the terrestrial plant samples (Figure 1).

**Figure 2:**
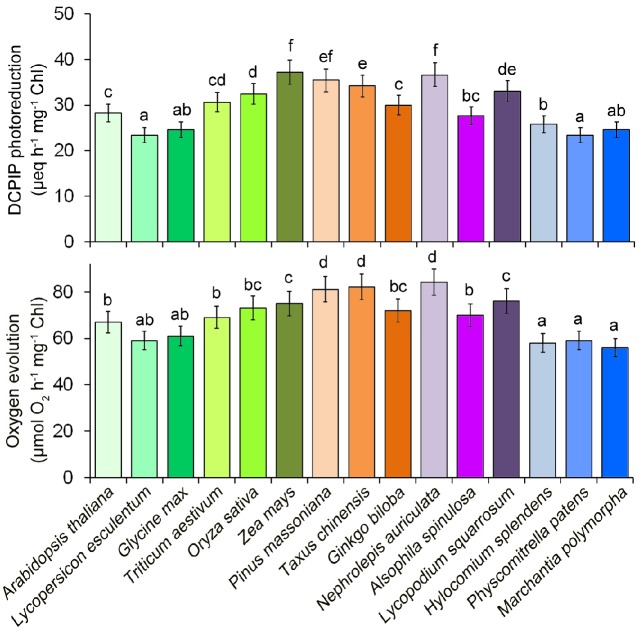
Photosystem II photochemical functions from 15 plant species. Oxygen evolution rates were measured with 0.25 mM phenyl-*p*-benzoquinone under saturating light intensities. DCPIP photoreduction was determined with 60 mM DCPIP. Values are expressed as the means ± SD from three independent biological replicates (*n* = 3), and values followed by different letters are significantly different at *p* < 0.05 according to Duncan’s multiple range test.

In a recent study, we provided an optimized method for extracting thylakoid membrane proteins (Chen et al., 2016b). However, it is unclear whether this optimized method is suitable for different plant species. To further test whether our previous optimized method for thylakoid extraction is suitable for different plants, photosynthetic oxygen-evolving activities and 2,6-dichlorophenol indophenol (DCPIP) photoreduction of the thylakoid membranes were compared among 15 different plant species (Figure 2). The results indicated that thylakoid membranes from all plants are functional and the thylakoid extraction method is still suitable for lower plants.

**Figure 3:**
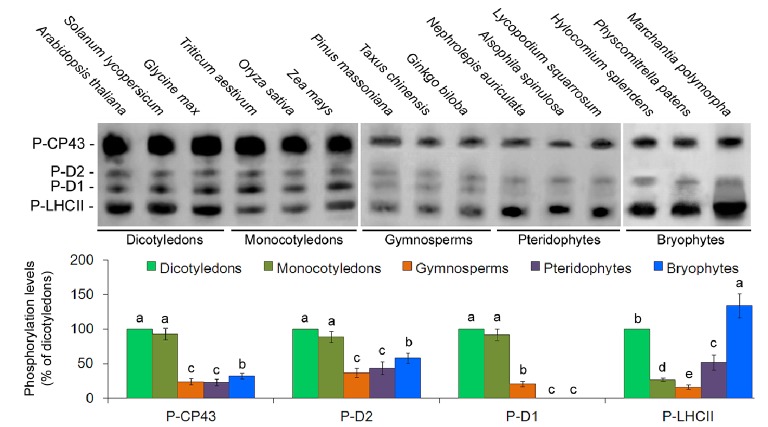
Thylakoid protein phosphorylation pattern in 15 plant species. PSII phosphoproteins were analyzed with antiphosphothreonine antibodies. Quantification of thylakoid protein phosphorylation is presented below the blots. Values are expressed as the means ± SD from three independent biological replicates (*n* = 3), and values followed by different letters are significantly different at *p* < 0.05 according to Duncan’s multiple range test.

To analyze the phosphorylation patterns of the thylakoid membrane proteins in evolutionarily divergent organisms, phosphoprotein-specific immunoblot experiments were carried out among the 15 different plant species using phosphothreonine antibodies. The main phosphoproteins were identified as P-CP43, P-D2, P-D1, and P-LHCII. However, no obvious D1 protein phosphorylation was detected in lower plants (Figure 3). Opposite that of the D1 protein, CP43, D2, and LHCII proteins were clearly phosphorylated in lower plants. Furthermore, the phosphorylation levels of the CP43 protein in angiosperms were stronger than in other species (Figure 3). Our results are in accordance with those of previous studies in *Ceratodon* (moss) and *Marchantia* (liverwort) (Pursiheimo et al., 1998).

### Separation of Thylakoid Protein Complexes by Improved BN-PAGE

*n*-dodecyl-β-D-maltoside (DM) is the most commonly used detergent for investigating thylakoid membrane complexes (Wittig et al., 2006; Järvi et al., 2011). The concentration of 0.5% DM was not sufficient to solubilize the thylakoid protein complexes, while 2% and 4% DM oversolubilized the thylakoid membrane, leading to a significant decrease in the amount of PSII-LHCII supercomplexes in *Arabidopsis*, wheat, and *Hylocomium* (Supplementary Figure S1). DM at 2% and 4% resulted in LHCII monomer binding with free pigments. Therefore, 1% DM was chosen as the optimal concentration. Surprisingly, the thylakoid membrane protein complexes could not be detected in BN-PAGE gels except for the LHCII trimer or LHCII monomer in both *Pinus* and *Nephrolepis* (Supplementary Figure S1). This may be because that the water solubility of thylakoid membrane proteins in either gymnosperms or ferns is low.

To improve the solubilization efficiency, two amphiphilic solubilizers (glycerol and polyethylene glycol) were added to the solubilization buffer with 1% (v/v) DM. Glycerol showed no obvious effects, while 5 and 10% polyethylene glycerol (PEG-6000) treatments with 1% DM resulted in the effective separation of thylakoid membrane protein complexes from *Pinus* and *Nephrolepis*, although 20% PEG-6000 showed negative effects (Supplementary Figure S2). The solubilization time had no effect on separation of membrane protein complexes (Supplementary Figure S3). The BN-PAGE bands of thylakoid membrane complexes from *Pinus* and *Nephrolepis* were further identified by the immunoblot assay (Supplementary Figure S4).

### Higher Plants Contain More PS Supercomplexes and LHCII Assemblies

**Figure 4:**
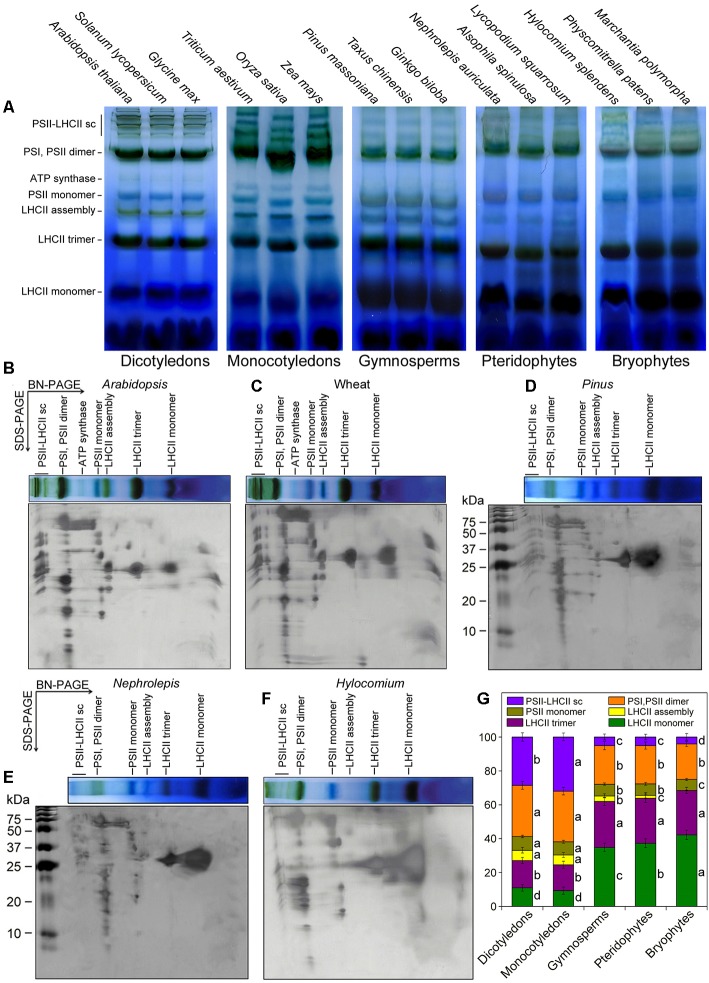
BN-PAGE analyses of thylakoid membrane protein complexes from 15 plant species. **(A)** BN-PAGE. Thylakoid membranes (20 μg of Chl) were solubilized with 1% DM in the presence of 5% PEG-6000 and separated by BN-PAGE. NDH, NAD(P)H dehydrogenase; mc, megacomplex; sc, supercomplex; Cyt, cytochrome. **(B–F)** Two-dimensional analyses of thylakoid membrane complexes from five typical plant species: *Arabidopsis thaliana* **(B)**, wheat **(C)**, *Pinus massoniana* **(D)**, *Nephrolepis auriculata* **(E)**, and *Hylocomium splendens* **(F)**. The BN-PAGE strips were then loaded horizontally onto the top of a 15% SDS-PAGE gel, and the gel was subsequently silver-stained. The molecular mass markers (in kDa) are as indicated. **(G)** The quantitative analysis of thylakoid membrane complexes. Values are expressed as the means ± SD from three independent biological replicates (*n* = 3), and values followed by different letters are significantly different at *p* < 0.05 according to Duncan’s multiple range test.

With the improved BN-PAGE method, we found the organization of thylakoid membrane protein complexes in 15 representative terrestrial plant species (Figure 4). Compared to dicotyledons, monocotyledons presented higher amounts of PSII-LHCII supercomplexes. In addition, the amounts of PSII-LHCII supercomplexes in three plant species (gymnosperms, pteridophytes, and bryophytes) were much lower than those in angiosperms. Gymnosperms, pteridophytes, and bryophytes also showed less PSII dimers relative to angiosperms. As opposed to angiosperms, LHCII monomers were more abundant than LHCII trimers in gymnosperms, pteridophytes, and bryophytes. Interestingly, bryophytes showed no LHCII assembly (LHCII supercomplexes). Although LHCII assembly occurred in pteridophytes and gymnosperms, their amount was much lower than that of angiosperms.

### Higher Chlorophyll Fluorescence and More Rapid Adaptation to Light Changes in Higher Plants

Chloroplast fluorescence is lower than the whole leaf fluorescence. Consistent with the smaller scale assembly of thylakoid protein complexes, lower plants displayed lower chloroplast fluorescence parameters *F*v/*F*m (the maximum quantum yield of PSII photochemistry), *Φ*_PSII_ (the actual quantum yield of PSII photochemistry), *Φ*_PSI_ (effective quantum yield of PSI photochemistry), and *Φ*_PSI (ND)_ (oxidation status of PSI donor side) (Table 1), demonstrating lower electron transport efficiencies from LHCII to the reaction centers (both PSII and PSI) in these plants.

**Table 1:** Chloroplast fluorescence parameters of 15 plant species.

Species		Chl *a* : Chl *b*	*F*_v_/*F*_m_	*Φ*_PSII_	*Φ*_PSI_	*Φ*_PSI (ND)_
Dicotyledons	*Arabidopsis thaliana*	2.99 ± 0.23^b^	0.78 ± 0.05^b^	0.31 ± 0.03^b^	0.41 ± 0.04^b^	0.37 ± 0.04^a^
	*Lycopersicon esculentum*	2.75 ± 0.21^c^	0.77 ± 0.05^b^	0.34 ± 0.03^b^	0.39 ± 0.04^b^	0.31 ± 0.03^b^
	*Glycine max*	2.78 ± 0.22^c^	0.82 ± 0.07^a^	0.39 ± 0.04^a^	0.45 ± 0.04^a^	0.39 ± 0.04^a^
Monocotyledonous	*Triticum aestivum*	3.17 ± 0.32^b^	0.79 ± 0.06^b^	0.35 ± 0.04^b^	0.43 ± 0.04^a^	0.38 ± 0.04^a^
	*Oryza sativa*	3.16 ± 0.32^b^	0.83 ± 0.07^a^	0.38 ± 0.04^a^	0.45 ± 0.04^a^	0.40 ± 0.04^a^
	*Zea mays*	3.79 ± 0.35^a^	0.85 ± 0.07^a^	0.39 ± 0.04^a^	0.47 ± 0.05^a^	0.43 ± 0.04^a^
Gymnosperm	*Pinus massoniana*	2.49 ± 0.20^d^	0.61 ± 0.04^e^	0.27 ± 0.03^c^	0.37 ± 0.03^c^	0.29 ± 0.03^b^
	*Taxus chinensis*	2.89 ± 0.21^c^	0.65 ± 0.05^d^	0.29 ± 0.02^c^	0.34 ± 0.03^c^	0.31 ± 0.03^b^
	*Ginkgo biloba*	2.80 ± 0.23^c^	0.71 ± 0.04^c^	0.28 ± 0.03^c^	0.36 ± 0.03^c^	0.30 ± 0.03^b^
Pteridophytes	*Nephrolepis auriculata*	2.03 ± 0.18^e^	0.66 ± 0.05^d^	0.24 ± 0.02^d^	0.31 ± 0.02^d^	0.27 ± 0.02^c^
	*Alsophila spinulosa*	2.33 ± 0.19^d^	0.75 ± 0.06^b^	0.23 ± 0.02^d^	0.32 ± 0.02^d^	0.28 ± 0.02^c^
	*Lycopodium squarrosum*	2.18 ± 0.19^d^	0.67 ± 0.05^d^	0.21 ± 0.02^d^	0.30 ± 0.03^d^	0.26 ± 0.03^c^
Bryophytes	*Hylocomium splendens*	1.98 ± 0.17^e^	0.63 ± 0.04^e^	0.21 ± 0.02^d^	0.30 ± 0.02^d^	0.27 ± 0.02^c^
	*Physcomitrella patens*	1.89 ± 0.17^f^	0.69 ± 0.04^c^	0.24 ± 0.02^d^	0.29 ± 0.02^d^	0.24 ± 0.02^d^
	*Marchantia polymorpha*	1.83 ± 0.16^f^	0.60 ± 0.04^e^	0.22 ± 0.02^d^	0.27 ± 0.02^e^	0.22 ± 0.02^d^

Values are expressed as the means ± SD from three independent biological replicates (*n* = 3), and values followed by different letters are significantly different at *p* < 0.05 according to Duncan’s multiple range test.

**Figure 5:**
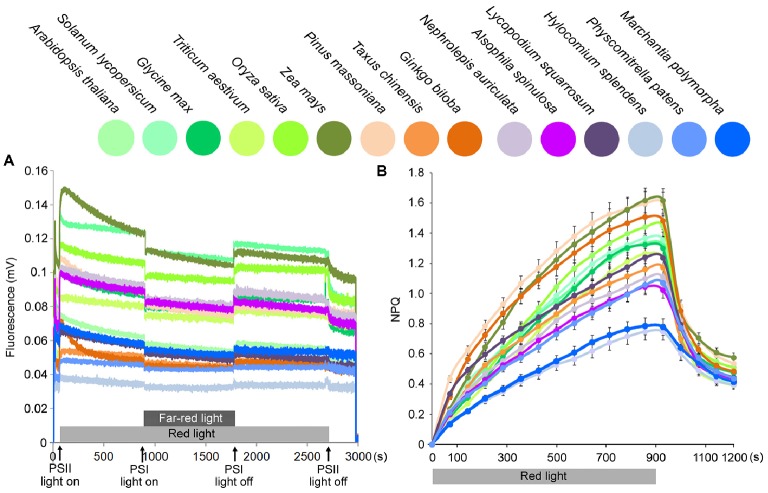
State transition and NPQ kinetics on chloroplasts from 15 plant species. **(A)** Analysis of state transition in 15 plant species. Chlorophyll fluorescence emission was measured upon treatment with red light and red light supplemented with far-red light, which induce transition to State 2 and State 1, respectively. **(B)** Measurements of NPQ kinetics on the chloroplasts from 15 plant species. Chloroplasts were illuminated with actinic red light of 1,000 μmol photons m^−2^ s^−1^ for 900 s. Symbols and error bars show means ± SD (*n* = 5).

An investigation of state transition (Figure 5A) revealed that, in higher plants, red light led to a greater increase in Chl fluorescence emitted from PSII, but then a rapid decline because of the energy redistribution to PSI. However, in lower plants, changes in light quality induced fewer changes in PSII fluorescence compared with higher plants (almost no state transition could be observed for bryophytes).

NPQ (non-photochemical quenching) kinetics was measured at a light intensity of 1,000 μmol photons m^−2^ s^−1^, and all plants displayed an initial increase and a subsequent decrease in NPQ. Lower plants showed decreases in the maximum NPQ values, especially in bryophytes (Figure 5B). A previous study indicated that *physcomitrella* NPQ is much stronger than *Arabidopsis* (Alboresi et al., 2010). However, they measured whole leaf NPQ. Here, in this study for chloroplast fluorescence, we found that maximum chloroplast NPQ values were much lower in bryophytes than in higher plants.

## Discussion

The poor solubilization of *Pinus* and *Nephrolepis* thylakoids might be because the water solubility of photosystem protein complexes in both gymnosperms and ferns is very low. Therefore, two amphiphilic solubilizers, glycerol and polyethylene glycol, were added to the solubilization buffer with 1% (v/v) DM. The different effects of glycerol and PEG-6000 on the separation of thylakoid membrane complexes may be because that PEG is a more effective amphiphilic solubilizer, causing lipid-soluble substances to dissolve in an aqueous solution as an aqueous emulsion (Demina et al., 2014).

It is well known that reversible D1 protein phosphorylation is considered one of the most important and genuine protective mechanisms against the irreversible damage of PSII through the PSII photoinhibition-repair cycle in higher plants (Tikkanen and Aro, 2012; Nath et al., 2013). In this report, no obvious D1 protein phosphorylation was detected in lower plants (Figure 3). A reason might be because the reversible phosphorylation of the D1 protein is the latest event in the evolution of PSII protein phosphorylation and occurs exclusively in seed plants—both gymnosperms and angiosperms (Pursiheimo et al., 1998). The fact that no antibody-detectable D1 protein phosphorylation was detected in pteridophytes and bryophytes may partly explain their slower Chl fluorescence changes in response to the light shift. Early studies with antiphosphothreonine antibodies claimed that cyanobacterial D1 protein is not a phosphoprotein (Pursiheimo et al., 1998), yet the new mass spectrometry analyses clearly indicate that indeed, also in cyanobacteria, the D1 protein is a phosphoprotein (Wang et al., 2014; Grieco et al., 2016). However, antibody-undetectable D1 phosphorylation (undetectable by the antiphosphothreonine antibody) in cyanobacteria might not be involved in the PSII photoinhibition-repair cycle as the higher plants do (Grieco et al., 2016), which requires further investigations.

The reasons for the difference in thylakoid protein complex organization may be due to plant evolution and their growth environments. Previous studies have indicated that the amounts of PSII-LHCII supercomplexes are low in shade plants (Pantaleoni et al., 2009; Kouřil et al., 2013; Ferroni et al., 2014). In the current study, our results showed that the low amounts of PSII-LHCII supercomplexes seemed to be a common phenomenon in gymnosperms, ferns, and mosses and LHCII assembly may be one of the evolutionary characteristics of vascular plants. Many studies have indicated that an obvious assembly and disassembly of the PSII-LHCII supercomplexes usually exists in higher plants in response to different environmental conditions (Albanese et al., 2016; Dall’Osto et al., 2017; Nosek et al., 2017). In general, disassembly of the PS supercomplexes (Chen et al., 2016a, 2017) and reduction of the functional PSII antenna size (Albanese et al., 2016) occur upon environmental stresses to avoid excess light energy and the subsequent ROS accumulation. And a recent report showed that the relative amount of LHCII monomers strongly increases in plants acclimated to high light, as the abundant amount of LHCII monomers under high light may be less efficient in transferring energy to the reaction center (Bielczynski et al., 2016), which have been also observed in lower plants (Figure 4).

No obvious correlation between thylakoid protein complex evolution and plastid genome organization and the coding capacity could be found. Most studies of land-plant chloroplast genomes focused on the gene introns (Daniell et al., 2016). For example, losses of introns within protein-coding genes have been reported in several plant species, such as barley, bamboo, cassava, and chickpea (Jansen et al., 2007). The proteins encoded by genes in which intron losses are known to occur have diverse functions, including an RNA polymerase subunit (rpoC2), an ATP synthase subunit (atpF), ribosomal proteins (rpl2, rps12, and rps16), and a Clp protease (clpP) (Jansen et al., 2007). However, they do not include the key genes encoding the PSII complex, such as psbA (encoding the D1 protein) and psbD (encoding the D2 protein). Therefore, terrestrial plants may evolve highly assembled photosystem complexes independent of plastid genome evolutions.

When plants are exposed to illumination favoring either PSII or PSI, they can redistribute excitation towards the light-limited photosystem. Changes in illumination lead to changes in photosystem stoichiometry (state transition; Lunde et al., 2000; Bellafiore et al., 2005). State 1 to State 2 transitions are active in limiting light conditions and inhibited in high light. Therefore, state transition is a dynamic mechanism that enables plants to respond rapidly to changes in illumination (Lunde et al., 2000; Bellafiore et al., 2005). In higher plants, PSII light led to a greater increase in PSII fluorescence, but then the exponential decline in fluorescence due to the energy redistribution to PSI. Less LHCII-PSI complexes that are formed in lower plants may partly explain their fewer changes in fluorescence during the state transition.

Although the exact mechanisms of feedback de-excitation (the qE type of NPQ) are not yet fully understood, it is clear that LHCII trimers are involved in some way and that the PsbS protein together with the xanthophyll cycle pigments have the capacity to catalyze the transition of the antenna from a nonquenched to a quenched state (Holt et al., 2005; Ahn et al., 2008). A recent study identified a mutant lacking all monomeric LHC proteins but retaining LHCII trimers and showed that NPQ induction rate in the mutant was substantially slower with respect to the wild type (Dall’Osto et al., 2017). This would be consistent with the fact that LHCII trimers would be fully competent to engage in qE quenching (Holt et al., 2005; Ahn et al., 2008), which are greatly compromised in lower plants.

**Figure 6:**
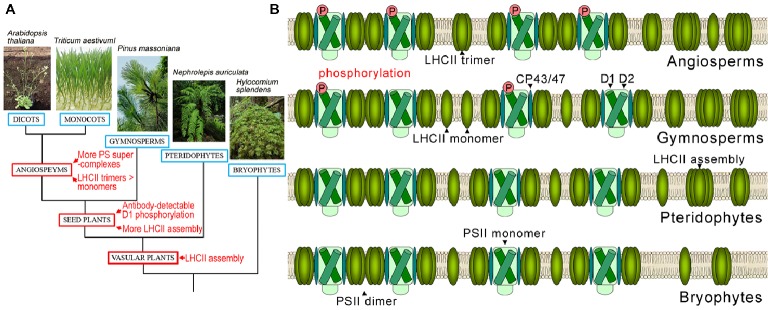
Evolutionary events of photosystem complexes in terrestrial plants. **(A)** Verbal description of evolutionary events of photosystem complex structures and D1 protein phosphorylation in terrestrial plants. **(B)** Schematic diagram of thylakoid PSII complex and LHCII complex structures in angiosperms, gymnosperms, pteridophytes, and bryophytes.

Our results imply that predominant PSII supercomplex assembly and increased LHCII trimer formation are the latest events in the evolution of photosystem complexes and exist exclusively in angiosperms (Figure 6). The high amounts of PSII supercomplexes in angiosperms may be correlated with their more efficient and more rapid responses to environmental changes.

## Author Contributions

Y-EC and SY designed the study. Y-EC, Y-QS, H-TM, NW, and FZ performed the research. Y-EC and SY wrote the paper. All the authors analyzed the data, discussed the results, and made comments on the manuscript.

### Conflict of Interest Statement

The authors declare that the research was conducted in the absence of any commercial or financial relationships that could be construed as a potential conflict of interest.
