# Supplementary material for: Terrestrial Plants Evolve Highly Assembled Photosystem Complexes in Adaptation to Light Shifts
Source: Front Plant Sci. 2018 Dec 19;9:1811. doi: 10.3389/fpls.2018.01811 (PMC6306036; doi:10.3389/fpls.2018.01811)
Supplement: Supplementary file 1 [file Data_Sheet_1.PDF]

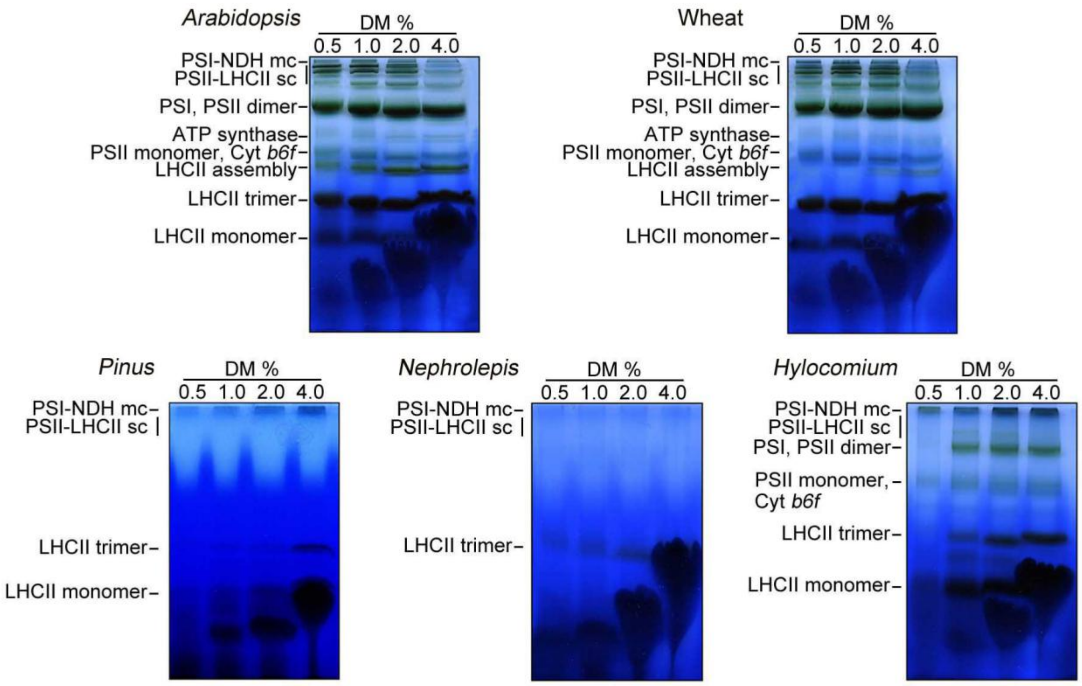

Supplementary Figure S1. Typical BN-PAGE analysis of thylakoid membrane protein complexes from *Arabidopsis thaliana*, wheat, *Pinus massoniana*, *Nephrolepis auriculata* and *Hylocomium splendens*.

Thylakoid membranes (20 µg of Chl) were solubilized with 0.5-4.0% (w/v) DM and separated by BNgel electrophoresis. NDH, NAD(P)H dehydrogenase; mc, megacomplex; sc, supercomplex; Cyt, cytochrome.

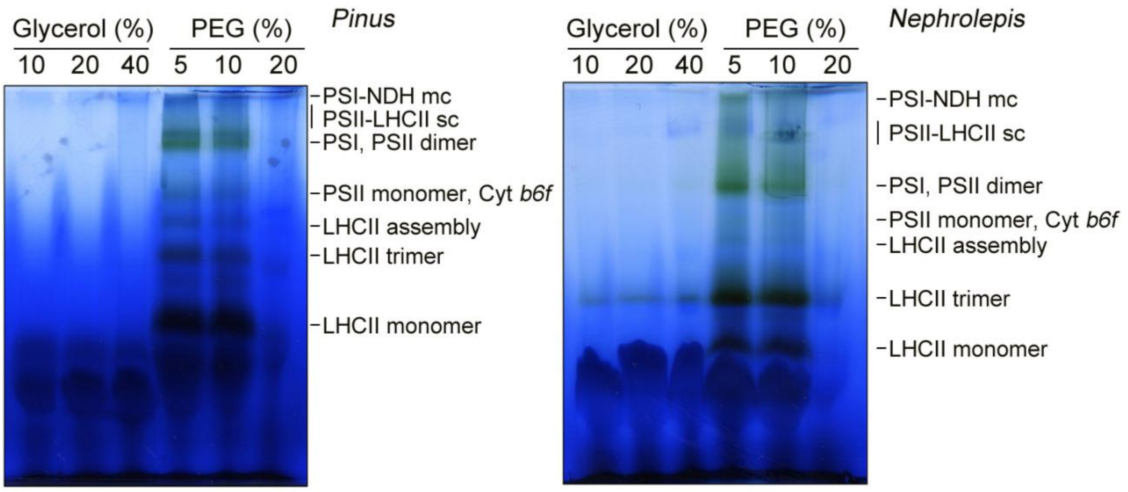

Supplementary Figure S2. BN-PAGE analyses of *Pinus* and *Nephrolepis* thylakoid membrane protein complexes solubilized with two lipophilic solvents at different concentrations.

Thylakoid membranes (20 μg Chl) were solubilized with 1% (w/v) DM in solubilization buffer with glycerol and PEG-6000 and were subjected to BN-PAGE. NDH, NAD(P)H dehydrogenase; mc, megacomplex; sc, supercomplex; Cyt, cytochrome.

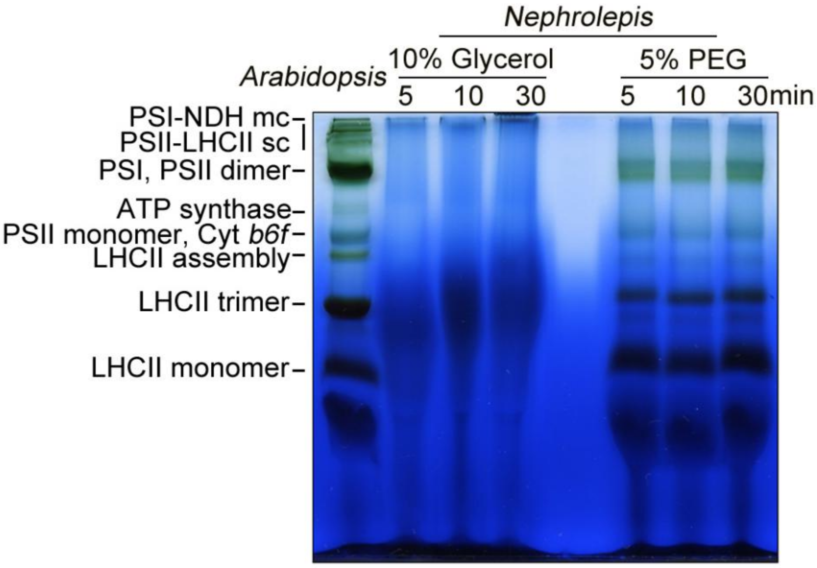

Supplementary Figure S3. BN-PAGE analyses of thylakoid membrane complexes solubilized with 1% (w/v) DM in the presence of 10% glycerol or 5% PEG-6000 for different times (5, 10, or 30 min) in *Nephrolepis*.

NDH, NAD(P)H dehydrogenase; mc, megacomplex; sc, supercomplex; Cyt, cytochrome. *Arabidopsis* was used as the indicator for the binding of thylakoid membrane protein complexes.

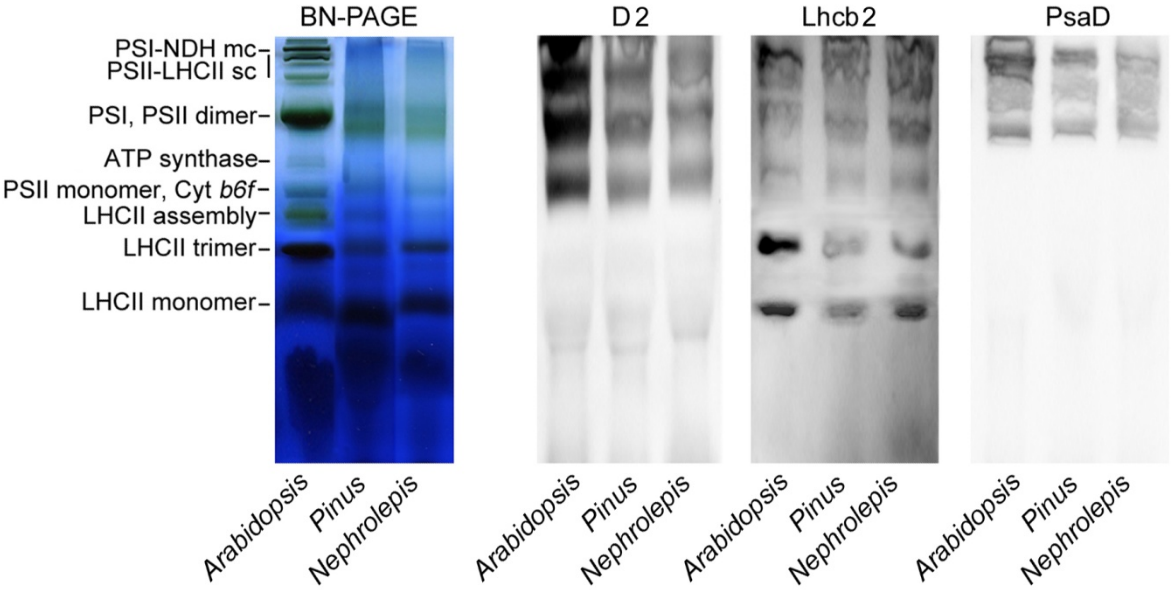

Supplementary Figure S4. Analysis and immunoblot determination of thylakoid membrane protein complexes from *Arabidopsis, Pinus* and *Nephrolepis*.

Thylakoid membranes (20 μg of Chl) were solubilized with 1% DM in the presence of 5% PEG-6000 and separated by BN-PAGE. NDH, NAD(P)H dehydrogenase; mc, megacomplex; sc, supercomplex; Cyt, cytochrome. The bands of BN-PAGE were confirmed by immunoblotting with D1-, Lhcb1- and PsaD-specific antibodies (on the right).
